# Modulating the activity of short arginine-tryptophan containing antibacterial peptides with N-terminal metallocenoyl groups

**DOI:** 10.3762/bjoc.8.200

**Published:** 2012-10-15

**Authors:** H Bauke Albada, Alina-Iulia Chiriac, Michaela Wenzel, Maya Penkova, Julia E Bandow, Hans-Georg Sahl, Nils Metzler-Nolte

**Affiliations:** 1 Inorganic Chemistry I – Bioinorganic Chemistry, Faculty of Chemistry and Biochemistry, Ruhr University Bochum, Universitätsstraße 150, 44801 Bochum, Germany; 2 Institute for Medical Microbiology, Immunology, and Parasitology, Pharmaceutical Microbiology Section, University of Bonn, Meckenheimer Allee 168, 53115 Bonn, Germany; 3 Microbial Biology, Faculty of Biology and Biotechnology, Ruhr University Bochum, Universitätsstraße 150, 44801 Bochum, Germany

**Keywords:** antimicrobial peptides, arginine, medicinal organometallic chemistry, metallocenoyl, peptides, tryptophan

## Abstract

A series of small synthetic arginine and tryptophan containing peptides was prepared and analyzed for their antibacterial activity. The effect of N-terminal substitution with metallocenoyl groups such as ferrocene (FcCO) and ruthenocene (RcCO) was investigated. Antibacterial activity in different media, growth inhibition, and killing kinetics of the most active peptides were determined. The toxicity of selected derivatives was determined against erythrocytes and three human cancer cell lines. It was shown that the replacement of an N-terminal arginine residue with a metallocenoyl moiety modulates the activity of WRWRW-peptides against Gram-positive and Gram-negative bacteria. MIC values of 2–6 µM for RcCO-W(RW)_2_ and 1–11 µM for (RW)_3_ were determined. Interestingly, W(RW)_2_-peptides derivatized with ferrocene were significantly less active than those derivatized with ruthenocene which have similar structural but different electronic properties, suggesting a major influence of the latter. The high activities observed for the RcCO-W(RW)_2_- and (RW)_3_-peptides led to an investigation of the origin of activity of these peptides using several important activity-related parameters. Firstly, killing kinetics of the RcCO-W(RW)_2_-peptide versus killing kinetics of the (RW)_3_ derivative showed faster reduction of the colony forming units for the RcCO-W(RW)_2_-peptide, although MIC values indicated higher activity for the (RW)_3_-peptide. This was confirmed by growth inhibition studies. Secondly, hemolysis studies revealed that both peptides did not lead to significant destruction of erythrocytes, even up to 500 µg/mL for (RW)_3_ and 250 µg/mL for RcCO-W(RW)_2_. In addition, toxicity against three human cancer cell lines (HepG2, HT29, MCF7) showed that the (RW)_3_-peptide had an IC_50_ value of ~140 µM and the RcW(RW)_2_ one of ~90 µM, indicating a potentially interesting therapeutic window. Both the killing kinetics and growth inhibition studies presented in this work point to a membrane-based mode of action for these two peptides, each having different kinetic parameters.

## Introduction

New antibacterial agents need to be discovered since established antibiotics are increasingly losing ground against resistant bacteria and at the same time the pipeline that is supposed to produce new antibiotics is running dry [1]. For example, the number of methicillin-resistant *Staphylococcus aureus* (MRSA) infections in hospitals are still very high and new infectious agents like *Acinetobacter baumannii* are on the rise, both leading to increased numbers of mortality. In view of this, the discovery of host-defense and antimicrobial peptides with bacteria-specific membrane targeting modes of action (MOA) to which resistance cannot easily develop has led to high expectations in the treatment of bacterial infections [2–4]. Whereas host-defense peptides are found in many multicellular organisms as part of their innate immune system, the name “antimicrobial peptides” (abbreviated as AMPs) defines a larger group of peptides that also encompasses synthetic peptides, and peptidomimetics, for example. Among these, synthetic peptide-based antimicrobial agents are especially interesting because isolation and/or synthesis of traditional organic molecules is often time-consuming and costly [3,5]. In fact, already in World War II, peptides belonging to a certain group of antimicrobial peptides, i.e., the gramicidins, found application in the treatment of gunshot wounds [6]. Unfortunately, their general toxicity prevented widespread systemic administration in the clinic. However, during the past couple of decades a large number of peptides with very potent antimicrobial activity and lower general toxicity were discovered [7].

Inspired by these antimicrobial peptides, many synthetic derivatives of naturally occurring antimicrobial peptides have been studied [5]. In addition, chemical syntheses of a large number of peptides that do not have natural counterparts have furnished promising synthetic antimicrobial peptides (synAMPs) [8]. For example, peptide-dendrimers [9–13], lipidated short peptides [14], trivalent lipidated short peptides with antifungal activity [15], peptoids [16], peptides containing D-amino acids [17], and foldamers based on β-amino acid residues with antibacterial activity [18] have been described. Whereas nature has to stick to products compatible with biosynthetic pathways, the synthetic chemist is free to apply all available compounds and techniques, thereby introducing even the most exotic molecular entities. The most recent and exotic additions are conjugates of metallocenes with short synthetic antimicrobial peptides [19–22] and organometallic derivatives of platensimycin [23–28].

Among the synAMPs known to date, those based on arginine (Arg or R) and tryptophan (Trp or W) residues are amongst the smallest peptides that still possess significant antibacterial activity. For example, Strøm et al. [29] described short RW-based synAMPs with different N- and C-terminal substituents, which showed low micromolar antibacterial activity against various strains of Gram-positive bacteria and moderate activity against Gram-negative bacteria. Interestingly, head-to-tail cyclized RW-based synAMPs with clustered functionalities increased the activity against Gram-negative *Escherichia coli* much more than against Gram-positive *Bacillus subtilis* [30–31], and only slightly increased the hemolytic activity [32]. Moreover, the alkylation of tryptophan residues by *tert*-butyl groups resulted in increased activity and low hemolytic activity of the constructs [33]. Our group has previously shown that the covalent attachment of metal complexes to RW-based synAMPs yields more active derivatives with a changed activity profile for Gram-positive and Gram-negative bacteria. In this work, the attachment of the neutral ferrocenoyl group (ferrocene: dicyclopentadienyl iron, Cp_2_Fe; ferrocenoyl: FcCO) was beneficial over the presence of the monocationic cobaltocenium (Cc^+^CO) fragment [20].

To gain a better understanding of the origin of the activity of these RW-based synAMPs and of the effect exerted on it by a metallocene moiety, a set of peptides was synthesized and tested for antimicrobial activity. In this paper we add another metal to the spectrum of existing organometallic synAMPs and we provide a detailed assessment of the kinetic parameters of this peptide. Specifically, we describe the effect of the introduction of ruthenocenoyl (ruthenocene: dicyclopentadienyl ruthenium, Cp_2_Ru; ruthenocenoyl: RcCO), an organometallic moiety that is almost isostructural to ferrocenoyl (FcCO) but has different electro- and physicochemical properties [34]. For example, the more extended d-orbitals of Rc form stronger hydrogen bonds with OH or NH groups than Fc [35]. The activities of the synAMPs (MIC values) were compared to those of GS(K_2_Y_2_) (Y = D-tyrosine), a gramicidin S analogue, and vancomycin, one of the last lines of defense against *Staphylococcus* infections. From the antibacterial activity screening, the two most active peptides were selected for further analysis, i.e., H-Arg-Trp-Arg-Trp-Arg-Trp-NH_2_ (referred to as (RW)_3_), and RcCO-Trp-Arg-Trp-Arg-Trp-NH_2_ (referred to as RcCO-W(RW)_2_). For these peptides, toxicity against three human cancer cell lines was assessed, followed by determination of their killing kinetics and growth inhibition potential. Please note that underlined one-letter codes of the amino acid residues represent D-amino acids, not underlined one-letter codes of the residues are L-amino acids.

## Results

### Synthesis of the synAMPs

**Figure 1:**
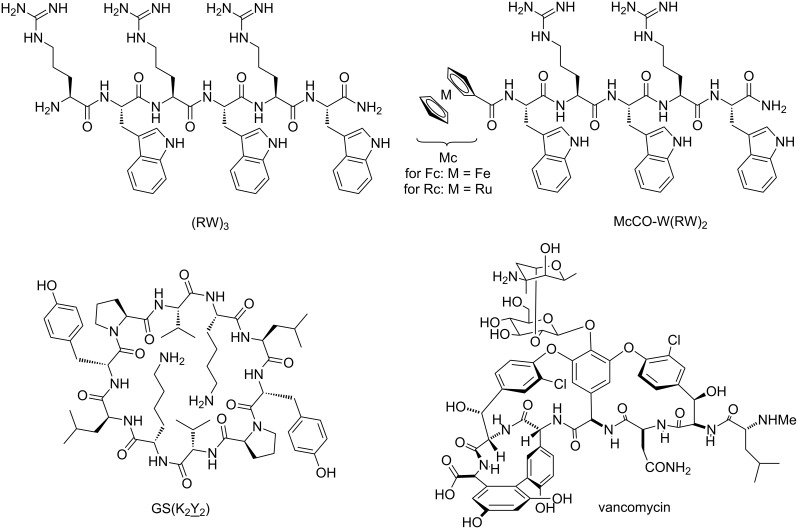
Structures of the most active peptides that have been used in this study. The top row shows two representative structures of the Arg-Trp based peptides (left) and their metallocene-derivatives (right); the lower row shows the structure of pore-forming gramicidin S derivative GS(K_2_Y_2_) (left) and lipid II-binding cell wall biosynthesis inhibitor vancomycin (right).

All peptides described in this study were prepared according to established or recently published procedures [36]. In short, Fmoc-protected amino acids were coupled in a solid-state synthesis scheme using HOBt, TBTU, and DiPEA under microwave irradiation. Using suitably protected amino acids, i.e., Fmoc-Arg(Pbf)-OH, Fmoc-Trp(Boc)-OH, and Fmoc-Lys(Boc)-OH, and polystyrene-based resin decorated with Fmoc-protected Rink linkers, a set of peptides were prepared (Table 1 and Figure 1). These peptides were obtained after acidic cleavage of the resin-bound protected precursors, purified by preparative HPLC, and the fractions containing the desired compound in high purity were lyophilized from the prep-HPLC buffers. All these peptides were obtained in high yields and close to 100% HPLC purity.

**Table 1:** Overview of the studied sequences and analysis thereof (retention times and *m*/*z* values). Underlined amino acids are D-enantiomers, not underlined residues are L-enantiomers; FcCO refers to ferrocenoyl and RcCO to ruthenocenoyl (Figure 1).

entry	sequence	*t*_R_ (min)	*m*/*z* found (calcd for [M + H]^+^)

1	H-RWRWRW-NH_2_	17.2	1044.25 (1044.58)
2	H-RWRWRW-NH_2_	17.2	1044.27 (1044.58)
3	RcCO-WRWRW-NH_2_	20.1	1146.27 (1146.44)
4	RcCO-WRWRW-NH_2_	20.1	1146.11 (1146.44)
5	FcCO-WRWRW-NH_2_	20.2	1100.36 (1100.47)
6	Ac-RWRWRW-NH_2_	17.6	1086.45 (1086.59)
7	Ac-RWRWRW-NH_2_	17.6	1086.37 (1086.59)
8	FcCO-RWRWRW-NH_2_	19.0	1256.48 (1256.57)
9	FcCO-RWRWRW-NH_2_	19.0	1256.46 (1256.59)
10	H-KWKWKW-NH_2_	16.7	959.43 (959.55)
11	vancomycin^a^	11.7	1448.56 (1448.44)
12	GS(K_2_Y_2_)^b^	25.0	1201.46 (1201.73)

^a^Vancomycin was obtained from Sigma-Aldrich Fluka and purified by preparative HPLC using a C_18_-reversered phase column; ^b^GS(K_2_Y_2_) = cyclo([Pro-Val-Lys-Leu-D-Tyr]_2_) was prepared according to [37].

### Biological activity

#### Minimum inhibitory concentration

The antibacterial activity of the peptides was first assessed by determining their minimum inhibitory concentration (MIC) value. This MIC value represents the lowest concentration of the antibacterial agent that is needed to hinder the growth of the bacteria [38]. For this, six standard bacterial strains – among them three Gram-negative and three Gram-positive pathogens – were incubated with increasing concentrations of the antibacterial peptide (Table 2). In order to put the activities of these RW-based synAMPs and their organometallic conjugates into perspective, two reference peptides were included, i.e., membrane-targeting gramicidin S derivative GS(K_2_Y_2_) and cell wall precursor lipid II-targeting vancomycin.

**Table 2:** Minimum inhibitory concentrations (µM) in the cell culture medium of the synAMPs described in this study (according to CSLI guidelines). Peptides have C-terminal carboxamides and are not derivatized on the N-terminus except where noted, i.e., with acetyl (Ac), FcCO or RcCO. Values in brackets are determined in Mueller–Hinton (MH) medium. More details on the bacterial strains can be found in the experimental section.

synAMP	Gram-negative			Gram-positive		
	*E. coli*	*A. baumannii*	*P. aeruginosa*	*B. subtilis*	*S. aureus*	*S. aureus* (MRSA)

(RW)_3_	21	21	n.a.	1.3	11 (11)	5.3 (11)
(RW)_3_	21	21	n.a.	1.3	5.3	5.3
(KW)_3_	n.a.	n.a.	n.a.	11–5.7	n.a.	n.a.
Ac(RW)_3_	45	–	–	45	–	–
Ac(RW)_3_	90	–	–	90	–	–
FcCO-(RW)_3_	20	–	–	20	–	–
FcCO-(RW)_3_	20	–	–	5	–	–
RcCO-W(RW)_2_	47	23–12	n.a.	2.9	5.8 (5.8)	5.8 (5.8)
RcCO-W(RW)_2_	23	23–11	93	1.5	2.9	1.5
FcCO-W(RW)_2_	>96	–	>96	12	–	48
vancomycin	76–38	38	n.a.	0.3	0.3	0.6
GS(K_2_Y_2_)	22–11	2.8–1.4	n.a.	1.4	1.4	2.8

For the calculations of the MIC values in µM, molecular weights of the peptides together with one TFA-counterion for each basic amino acid residue were used. ‘n.a.’ means ‘not active’ (MIC > 100 μM), ‘–‘ indicates that these MIC values were not determined.

In general, the activity of these synAMPs against Gram-negative bacteria is lower than against Gram-positive pathogens. Even if differences can be seen in the Gram-negative values, none of the RW-peptides was very active. Unfortunately, none of the peptides showed significant activity against *Pseudomonas aeruginosa*, a prominent pathogen that causes infections in e.g., cystic fibrosis patients. However, activities of the synAMPs against Gram-positive bacteria are only slightly lower than those of gramicidin S derivative GS(K_2_Y_2_), a peptide that contains twice as many amino acids as RcCO-W(RW)_2_. Interestingly, the replacement of the acetyl-group in Ac(RW)_3_ with the ferrocenoyl moiety results in more active peptides, which is most likely due to the increased hydrophobicity of the FcCO-peptide (*t*_R_ 17.6 min (for Ac(RW)_3_) vs 19.0 min (for FcCO-(RW)_3_). Although hydrophobicity seems to be important for the activity of these synAMPs, it is not the dominant factor in the organometallic derivatives. For example, replacement of the ruthenium atom with iron, going from RcCO to FcCO, results in a 4-fold and 8-fold drop in activity against *B. subtilis* and *S. aureus* (MRSA), respectively, even though their hydrophobicity is very similar. Since Rc is slightly larger than Fc – i.e., metal–carbon bonds in the first are 221 ppm whereas those in the latter are 204 pm [39–40], a difference of about 0.17 Å – the difference in size of the two metallocene derivatives could contribute to the significant difference in activity. In addition, it has been described that ruthenocene is a stronger hydrogen bond acceptor than ferrocene [37], which originates from more extended d-orbitals of the Rc when compared to Fc [41].

The comparable activities of (RW)_3_ and RcCO-W(RW)_2_ are especially remarkable since the peptides have very different properties, i.e., the first peptide has four positive charges and three units of lipophilic bulk (*t*_R_ = 17.2 min) whereas the second peptide has only two positive charges and four units of lipophilic bulk (*t*_R_ = 20.1 min). Whereas it is known that tryptophan residues function as membrane anchors [42], details of the interaction between metallocene derivatives and bacterial membranes are far from being understood. Importantly, the activity of these peptides was comparable in two different media, namely the bacterial Mueller–Hinton (MH) and in the richer cell culture medium. Interestingly, replacement of the arginine residues with lysine residues resulted in an almost completely inactive (KW)_3_ peptide [43]. Although the center of the positive charge in both residues is found at five atoms from the backbone, the different structures of the functional groups and the hydrophobicity of the side chain seem to cause a significant difference in activity [44–45].

From the initial screening, four peptides were selected for further biological characterization. In view of the mentioned differences in structure, including the addition of a novel metal core in one of them, but rather similar activity the (RW)_3_ and RcCO-W(RW)_2_ peptides were chosen. Since both L- and D-amino acid versions of the (RW)_3_ and RcCO-W(RW)_2_ peptides are comparable in activity and represent the most promising synAMPs (Table 2), we next determined the MIC values of these four peptides against other Gram-positive bacteria in order to expand the panel of test strains (Table 3).

**Table 3:** Detailed assessment of the MIC values (in µg/mL) of both L- and D-versions of the (RW)_3_ and RcCO-W(RW)_2_ synAMPs against several Gram-positive bacterial strains. More details on the bacterial strains can be found in the experimental section.

strain	(RW)_3_	(RW)_3_	RcCO-W(RW)_2_	RcCO-W(RW)_2_

*S. aureus* (133)	2.1 ± 0.7	2.1 ± 0.7	3.3 ± 1.4	6.7 ± 2.9
*S. simulans* (22)	5.0 ± 0.0	3.3 ± 1.4	5.0 ± 0.0	6.7 ± 2.9
*S. aureus* (SG511)	6.7 ± 2.9	4.2 ± 1.4	4.2 ± 1.4	8.3 ± 2.9
*B. subtilis*	3.5 ± 1.8	3.8 ± 1.8	3.8 ± 1.8	3.8 ± 1.8
*B. megaterium*	0.8 ± 0.7	0.5 ± 0.2	1.9 ± 0.9	2.5 ± 0.0
*M. luteus*	0.6 ± 0.0	0.6 ± 0.0	0.9 ± 0.4	1.9 ± 0.9

As can be inferred from this table, the L- and D-amino acid versions of these two synAMPs show comparable activity although small differences can be observed. For example, RcCO-W(RW)_2_ is almost twice as active against *S. aureus* (SG511) as the D-amino acid isomer RcCO-W(RW)_2_, a difference that can be seen as a tendency against all but one of the bacterial strains. Since the biological world is chiral, it is not surprising to see some small differences between both chiral forms of the synAMPs. Similar differences in the interaction with chiral molecules and biological membranes have been described before [46–48], although only in a few examples and not without exceptions [49]. An opposite trend is seen for the (RW)_3_-peptides, where the D-peptides show higher MIC values than the L-peptides. These values could have been corroborated, however, by preferential proteolytic degradation of the N-terminally unprotected (RW)_3_-peptides [50].

In order to obtain more information on the antibacterial properties of these four peptides, we performed killing kinetics and growth inhibition studies. Finally, we assessed the selectivity of the RcCO-W(RW)_2_ and (RW)_3_ peptides towards bacteria by determining their activity against human red blood cells and several human cancer cell lines.

#### Killing kinetics

**Figure 2:**
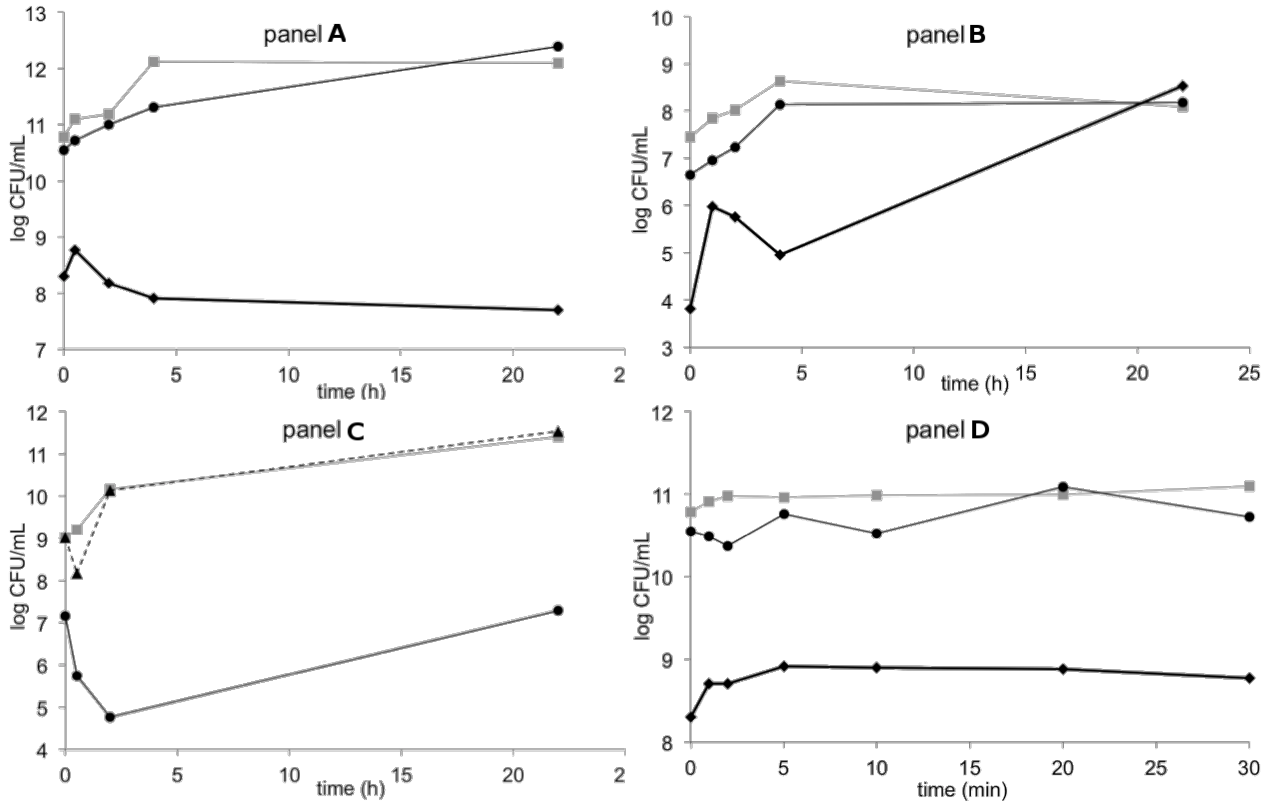
Bactericidal activity of (RW)_3_ against *S. aureus* 133 (panel **A** and **D**) or *B. megaterium* (panel **B**) and of RcCO-W(RW)_2_ (panel **C**) against *S. aureus* 133. Panel **D** shows the experiment on *S. aureus* 133 (as in panel **A**) using short-term intervals for sample collection (note that the time-scale is given in minutes). The first points in each graph are obtained after 1 min. Concentrations are denoted by: grey squares (for the control), black triangles with dotted line (only in panel **C**, 0.5 × MIC: 1.0 µg/mL for RcCO-W(RW)_2_), black circles with narrow line (1 × MIC: 0.8 µg/mL for (RW)_3_ and 1.9 µg/mL for RcCO-W(RW)_2_), black diamonds with thick line (5 × MIC: 4 µg/mL for (RW)_3_, only in panels **A**, **B**, and **D**).

Killing kinetics experiments show the rate at which bacteria are killed over time and indicate whether an antibacterial agent has a bacteriostatic or bactericidal activity. The killing kinetics of the L-amino acid containing (RW)_3_- and RcCO-W(RW)_2_-peptides were determined against *S. aureus* and *B. megaterium* (Figure 2).

For this, peptides were added in various concentrations to bacterial cultures at the optical densities of 0.1 at OD_600_. Aliquots of the mixture were taken at given time points, plated in duplicate on MH agar and incubated at 37 °C. Then, the number of colony forming units (CFU) was counted (see Experimental section for details).

The addition of the (RW)_3_ peptide to the bacterial culture resulted in a strong inhibition and in an immediate reduction of CFUs by a factor of 10^3^ after 1 min, for both *S. aureus* and *B. megaterium*. Similarly, treatment with RcCO-W(RW)_2_ also decreased the number of CFUs and has shown increased potency since only one dose at the MIC value was needed to decrease the CFUs by 2–3 log units, whereas 5 × MIC of (RW)_3_ was required for a similar drop of CFUs. The immediate drop in CFUs highlights the bactericidal nature of these synAMPs and typically occurs with membrane acting compounds [51–52].

#### Growth inhibition

Whereas the killing kinetics studies determine the number of viable cells as a function of time and thereby classify a compound as bacteriostatic or bactericidal, monitoring the optical density of a treated culture may give hints as to the lytic activity of a compound.

**Figure 3:**
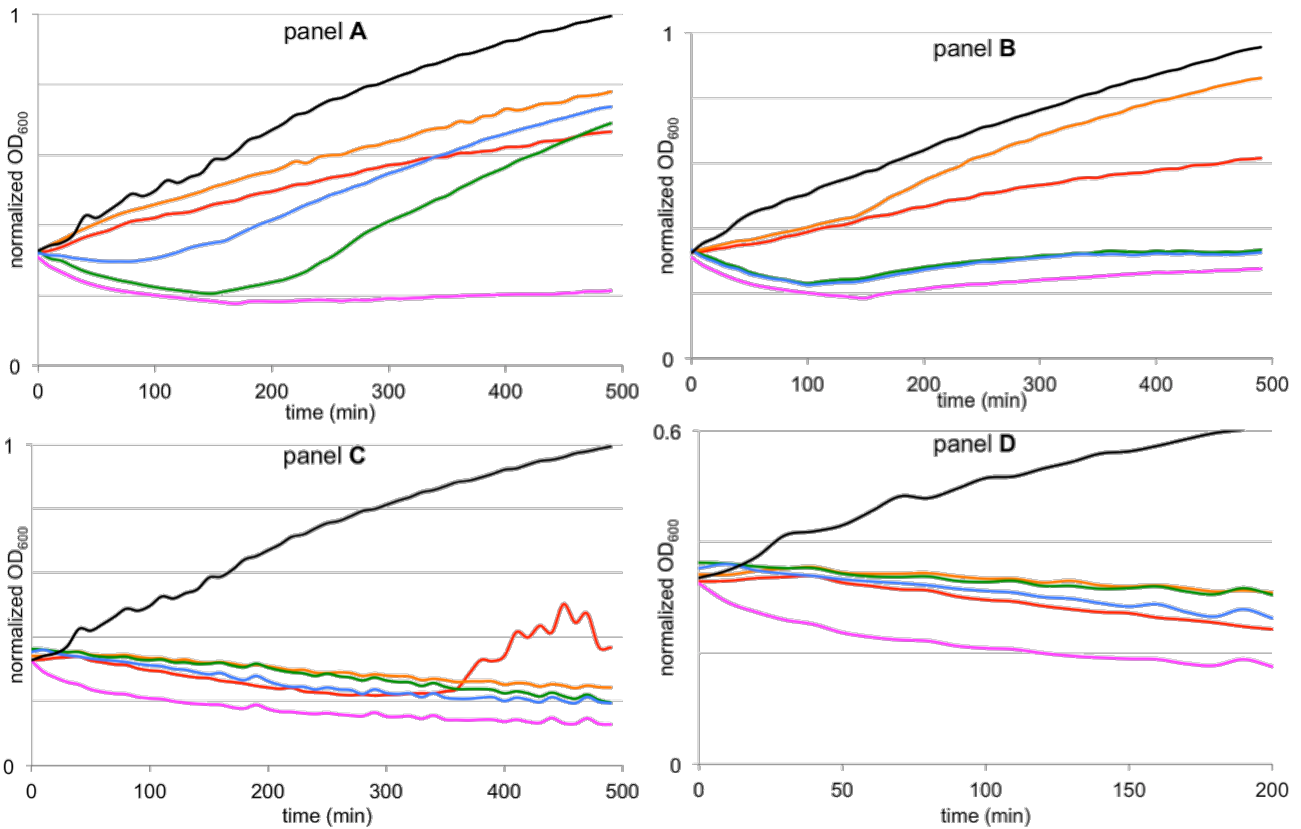
Growth kinetics of *B. megaterium* under the influence of different amounts of synAMP (red: (RW)_3_; orange: (RW)_3_; green: RcCO-W(RW)_2_; blue: RcCO-W(RW)_2_), nisin (magenta), and a control (black). With 2 × MIC (1.6 µg/mL for (RW)_3_ and 3.8 µg/mL for RcCO-W(RW)_2_ panel **A**), 4 × MIC (3.2 µg/mL for (RW)_3_ and 7.6 µg/mL for RcCO-W(RW)_2_; panel **B**, note: The green line for RcCO-W(RW)_2_ is under the blue line for RcCO-W(RW)_2_), and 8 × MIC (6.4 µg/mL for (RW)_3_ and 15.2 µg/mL for RcCO-W(RW)_2_; panel **C** and **D**). Gridlines at 0.2, 0.4, 0.6, and 0.8 normalized OD_600_ are shown.

In this work, the growth inhibition of *Bacillus megaterium* was determined under the influence of the same four peptides used in the killing kinetics studies described earlier (Figure 3). To determine this, *B. megaterium* was grown in half Mueller–Hinton (MH) medium and stressed with peptides (RW)_3_ or RcCO-W(RW)_2_ in their exponential growth phase. Three concentrations were used: 2 × MIC, 4 × MIC and 8 × MIC. As a positive control the potent naturally occurring pore-forming lantibiotic nisin was included.

At twice the MIC value, both of the D-amino acid containing derivatives are slightly inferior for inhibiting growth than the L-amino acid variants and the RcCO-W(RW)_2_*-*peptides are more active than the (RW)_3_-peptides (Figure 3). This is in line with our earlier observation, i.e., that the interaction of the bacterial target and D-peptides is slightly less favorable than that with L-peptides. Interestingly, the growth inhibition is more efficient with the RcCO-W(RW)_2_ peptides than with the (RW)_3_ peptides (Figure 3, panel **A**), although the cells treated with the RcCO-W(RW)_2_ peptide recover faster than those treated with the (RW)_3_ peptide. This is also reflected in the similar MIC values observed. It appears that the RcCO-W(RW)_2_ peptides are faster acting than the (RW)_3_ peptides but may produce cellular stress that can be better overcome by survivors in the course of an MIC determination experiment (which takes about 18 h). This difference in growth inhibition between the metalated and non-metalated peptide is even more pronounced at four times the MIC value. At this concentration it seems that the Rc-derivatized synAMPs are much more active than the non-derivatized counterparts (Figure 3, panel **B**). Moreover, at this concentration the RcCO-W(RW)_2_-peptides display the same inhibition potency as nisin whereas the (RW)_3_ peptides are much less active. At eight times the MIC value the bacterial growth inhibition was accompanied by cell lysis. For all compounds, the effect was equally strong so that a clear distinction could not be observed anymore and all the peptides exhibited the same effect on bacterial growth as nisin (Figure 3, panels **C** and **D**). These findings are in agreement with the killing kinetics in that RcCO-W(RW)_2_ is faster in killing than (RW)_3_.

#### Hemolytic activity against human red blood cells

After this, we assessed the hemolytic properties of these four most active peptides (Table 4). Although an HC_50_ value was reported for (RW)_3_ [53], neither the D-amino acid peptide nor the organometallic derivatized peptides were studied with respect to their hemolytic capacity. Therefore, all peptides were tested in parallel to obtain HC_50_ values under identical conditions.

**Table 4:** Hemolytic activity of both L- and D-peptides of the (RW)_3_ and RcCO-W(RW)_2_ synAMPs against human red blood cells (hRBCs).

synAMP	hemolytic activity

(RW)_3_	17% hemolysis at 500 µg/mL (333 µM)
(RW)_3_	0% hemolysis up to 500 µg/mL (333 µM)
RcCO-W(RW)_2_	64% hemolysis at 263 µg/mL (192 µM)
RcCO-W(RW)_2_	68% hemolysis at 263 µg/mL (192 µM)

As can be seen from these results, none of these peptides is strongly hemolytic. For example, each of the two (RW)_3_ compounds showed less than 50% hemolysis up to 500 µg/mL (333 µM). This value is higher from what has been reported before by Liu et al. (who reported 50% hemolysis at ~250 µM [53]). In fact, only the L-amino acid peptide (RW)_3_ showed weak hemolysis at 333 µM with 17% of the hRBCs being destroyed as compared to Triton X-100. These low hemolytic properties for both (RW)_3_-peptides, even up to 500 µg/mL, did not allow us to calculate their HC_50_-values.

The high concentrations of the ruthenocene derivatives required 50% DMSO/PBS-buffer mixtures for solubility. Using the appropriate blanks we found that >60% of the hRBCs were lysed using 195 µM of the peptide, with RcCO-W(RW)_2_ being more active than its L-amino acid counterpart. Using these directly observed values, approximate HC_50_ values of 153 µM (or 210 µg/mL) and 143 µM (or 196 µg/mL) were calculated for RcCO-W(RW)_2_ and RcCO-W(RW)_2_, respectively.

Thus, the organometallic ruthenocenoyl-conjugated synAMPs are more hemolytic than the parent (RW)_3_-peptides. Moreover, whereas the L-amino acid version of (RW)_3_ was more active that the peptide containing only D-amino acids, the opposite was observed for the two RcCO-W(RW)_2_ peptides. Nevertheless, none of the obtained values showed strong hemolytic activity of either of these peptides. This encouraged us to go ahead and test the activity of these peptides against several human cancer cell lines in order to assess in vitro cell-toxicity.

#### Toxicity against human cancer cell lines

Finally, to determine whether the peptides are selective for bacterial cells, we tested the toxicity against mammalian cells using three malignant cell lines: human liver carcinoma (HepG2), human colon adenocarcinoma grade II (HT29) and human breast adenocarcinoma (MCF7) cell lines (Table 5).

**Table 5:** IC_50_ values (in µM) of both (RW)_3_ and RcCO-W(RW)_2_ against human liver carcinoma (HepG2), human colon cancer (HT29) and human breast cancer (MCF7) cell lines.

synAMP	HepG2	HT29	MCF7

(RW)_3_	143 ± 21	132 ± 12	159 ± 7
RcCO-W(RW)_2_	92 ± 5	94 ± 6	90 ± 1

In general, we consider a peptide with an IC_50_ value higher than 100 µM to be inactive. As can be seen from Table 5, the peptides with the highest activity against Gram-positive bacteria are not toxic against the three selected human cancer cell lines. Based on average values from these cell lines, i.e., 142 μM for (RW)_3_ and 92 μM for RcCO-W(RW)_2_, a potential therapeutic window of about 7 and 4 can be calculated for Gram-negative pathogens using (RW)_3_ and RcCO-W(RW)_2_, respectively. Concerning the threatening Gram-positive *S. aureus* strains an even better window of >13 is calculated for (RW)_3_ and RcCO-W(RW)_2_. Interestingly, again the ruthenocenoyl-derivatized synAMP is more active than the (RW)_3_ model peptide, as was seen in both the antibacterial and hemolysis studies.

## Discussion

Ruthenium is one of the most promising metals in anticancer drug candidates [54–57], with two Ru-compounds even in clinical trials [58–62]. Surprisingly however, its potential in antibacterial research has not been explored so far. In this paper, we present the effects of the attachment of the organometallic ruthenocene (Rc) moiety to RW-based synthetic antimicrobial peptides (synAMPs). A comparison of the MIC values from a first screening of peptides that were N-terminally derivatized with a ruthenocenoyl (RcCO) group with that of the ferrocenoyl (FcCO)-derivatized peptides showed superior properties of the Rc-conjugated synAMPs (Table 2). Although both metallocenes have very similar hydrophobic properties, as confirmed herein again by their almost identical retention times on a C_18_-column during HPLC-analysis, they have slightly different dimensions and very different physicochemical and electrochemical properties. Firstly, ferrocene derivatives have redox potentials that are within the realm of biological systems, but ruthenocene derivatives do not [41]. Secondly, while most ferrocene derivatives exhibit a reversible one-electron oxidation, ruthenocene and its derivatives typically undergo irreversible two-electron redox chemistry. Whether this difference in redox chemistry of the two metallocenes could interplay with the piezoelectric properties of phospholipid membranes [63] remains to be determined.

Ruthenocene is known to have more extended d-orbitals and is a stronger hydrogen-bond acceptor than ferrocene. In addition, ruthenocene is slightly larger than ferrocene, which might result in a possibly more disruptive interaction with bacterial membranes.

Small differences between the L- and D-amino acid versions of the peptides could be observed in their MIC values (Table 3) and within growth inhibition studies (Figure 3). Examples for this difference were found in other systems (see above) and points to a delicate contribution of the chirality of the peptides used. This effect was not observed in the first MIC values determined (Table 2), which indicates that this is a very subtle factor. Indeed, the quantification requires a more sophisticated analysis. This can be done using sensitive biophysical model systems like those used in quartz crystal microbalance (QCM) studies. This information can then be used to further optimize an active synAMP.

Fortunately, while retaining antibacterial activity in cell culture medium, the cellular toxicity of both the (RW)_3_ and the RcCO-W(RW)_2_ peptides is low, and only high peptide concentrations cause significant hemolysis. Apparently, these peptides have a strong preference for prokaryotic membranes over eukaryotic membranes, e.g., erythrocytes (Table 4 and Table 5). Nevertheless, it remains to be seen to what extent these short synAMPs can be used in vivo.

Concerning the antibacterial effect of these peptides, the killing kinetics showed rapid bactericidal properties of both the (RW)_3_ and RcCO-W(RW)_2_ peptides, and the growth kinetics showed growth arrest and also indicated bacteriolytic properties. Naturally occurring AMPs such as nisin and magainin typically have >20 amino acids and often have a specific target, like nisin, or are long enough to penetrate the membrane, like magainin. For these long peptides descriptions of their action mode with the “carpet-model”, the “toroidal pore model” or the “barrel stave model” [64] are quite suitable. Considering the rapid upon-contact killing and bacteriolytic properties, it appears that the small synAMPs studied herein interact with the bacterial membrane as well. The monomers of these peptides are, however, too short to penetrate a bacterial membrane in order to form pores, and therefore, probably act slightly different from the more or less well-established mechanisms for longer AMPs. We are currently undertaking efforts to uncover more details on the mode of action. Specifically, proteomic analysis of the changes in the bacterial proteome as result of exposure to these synAMPs, and prokaryotic and eukaryotic membrane model systems will be used to precisely determine if it is simply a membrane-based mechanism or if there are more factors. While we attempt to elucidate the mode of action of these synAMPs, we are also interested in a detailed understanding of the effects of the organometallic fragment on the activity – for example by determining the contributions of hydrogen-bond forming processes in membrane environments – and the effect of the redox potential on the activity. We assume that the application of model systems will help us to determine the extent in which differences in chirality of the amino acids used to construct the peptides result in more or less favorable interactions.

## Conclusion

We have shown that the replacement of the N-terminal arginine residue in non-toxic and non-hemolytic (RW)_3_ peptides can modulate the kinetics of the peptide’s antibacterial activity. Acetylation completely suppresses this activity. In comparison, replacement of the N-terminal arginine residue with the organometallic ferrocenoyl moiety reduces the activity only 5- to 10-fold, whereas the replacement with ruthenocene completely restores the level of activity. In summary, the data supports a metal-specific activity-enhancing effect of the added organometallic moiety. This effect is most likely due to the added lipophilic bulk together with intricate contributions from the electro- and physicochemical properties of the organometallic fragment. None of these peptides is hemolytic and both are hardly toxic against human cancer cell lines. Thereby, they represent an interesting group of synthetic antimicrobial peptides to be used in a therapeutic setting. Analysis of the antibacterial properties of these peptides showed that they are rapidly bactericidal and also bacteriolytic. Even though both peptides have similar MIC values, RcCO-W(RW)_2_ is acting faster than (RW)_3_, but is losing activity after 100–200 min, which is significantly faster compared to (RW)_3_. Future studies on these peptides are directed towards a better understanding of their mode of action and attempts are being made for the improvement of their activity to increase the therapeutic window of these compounds.

## Experimental

### Minimal inhibitory concentration (results are shown in Table 2)

The minimal inhibitory concentrations (MIC) were tested against *Escherichia coli* DSM 30083, *Acinetobacter baumannii* DSM 30007, *Pseudomonas aeruginosa* DSM 50071, *Bacillus subtilis* DSM 402, *Staphylococcus aureus* DSM 20231 (type strain), and *Staphylococcus aureus* ATCC 43300 (MRSA) in a microtiter plate assay according to CLSI guidelines [65]. *E. coli*, *A. baumannii*, *S. aureus*, and *B. subtilis* were grown in Mueller–Hinton (MH) broth, *P. aeruginosa* in cation adjusted Mueller–Hinton II. Peptides were dissolved in DMSO to give 10 mg/mL stock solutions. Serial dilution in culture media was carried out automatically with the Tecan Freedom Evo 75 liquid handling workstation (Tecan, Männedorf, Switzerland) from 512 to 0.5 µg/mL. Peptide dilutions were inoculated with 10^5^ bacteria/mL taken from late exponential cultures grown in the same media in a total volume of 200 µL per well. Cells were incubated for 16 h at 37 °C. The lowest peptide concentration inhibiting visible bacterial growth was taken as MIC.

For MIC determination in cell culture broth, the peptides were diluted manually in DMEM high glucose (with 4.5 g/L glucose, no penicillin). Only *S. aureus* DSM 20231 and ATCC 43300 were capable of growing in cell culture broth and were used for MIC determination. Cells were grown in DMEM until late exponential phase before using them for inoculation. Peptide concentrations, inoculation and incubation were performed as described above.

### Minimum inhibitory concentration (results are shown in Table 3)

Determination of MIC values was performed in 96-well polypropylene microtiter plates (Life Technologies) in order to reduce the AMP binding [66]. A series of 2-fold dilutions of the peptides was prepared directly in the plate in half-concentrated MH broth. The tested strains were grown to an optical density (600 nm) of 0.5 in half-concentrated MH broth and diluted 1:10^5^ using the same medium. Then, 100 µL of this suspension was mixed with 100 µL of the peptide solution already prepared in the wells of the microtiter plate as mentioned earlier. After incubation for 18 h at 37 °C, the MIC value was read as the lowest concentration of antimicrobial agent that resulted in complete inhibition of visible growth. The results given are mean values of three or more independent determinations.

### Killing kinetics

The cells were grown in half-concentrated MH broth up to an optical density of 0.5 and diluted in fresh medium to an optical density of 0.1. Peptides were added in concentrations corresponding to 0.5 to 5 × MIC. The viable count was monitored up to 18 h. Aliquots were taken at defined time intervals, diluted in 10 mM potassium phosphate buffer, and 100 µL of several decimal dilutions were plated in duplicate on MH agar. The plates were incubated at 37 °C and the plates containing 30–300 colony forming units (CFU) were counted after 24 h.

### Kinetic growth inhibition

Growth kinetic experiments were performed in microtiter plates using 200 µL half concentrated MH broth. The cells were grown to an optical density of up to 0.5 and diluted in fresh medium to an optical density of 0.25. After this, peptides were added in concentrations corresponding to 2 × MIC, 4 × MIC, and 8 × MIC and the optical density was registered for 8 h using a multichannel absorbance plate reader (SunriseTM, Tecan).

### Hemolysis and in vitro cell toxicity studies

After drawing whole blood into anticoagulant containing tubes (BD Vacutainer^®^, K2 EDTA 3.6 mg, Ref 368841, Lot 1248213), its fractionation was executed with one volume whole blood added to nine volumes sterile 0.9% NaCl and centrifugation (800*g*, 10 min, 4 °C). Subsequently, the lowest fraction containing all hRBCs was washed twice with nine volumes 1 × PBS (PAA), triturating carefully. The concentrated hRBCs were re-suspended with 1 × PBS to an erythrocyte concentration of 5% (v/v). Wells of a 96-well plate were filled with 100 µL of the appropriate peptide solutions: The peptides were dissolved in 1 × PBS and DMSO (5% for (RW)_3_ and 50% for RcCO-W(RW)_2_). These were mixed with 100 µL of the 5% hRBCs solution and incubated under agitation on a flat shaker (170 rpm, 30 min, 37 °C). After sedimenting all probes under centrifugation (800*g*, 10 min, 4 °C), all supernatants were transferred into a clean 96-well plate. The release of hemoglobin was monitored by measuring the absorbance of the supernatant at 550 nm using an automated 96-well plate reader. Controls for 0 and 100% hemolysis consisted of hRBC 5% (v/v) suspended in PBS containing DMSO in appropriate concentrations and 1% Triton X-100, respectively. Toxicity on human cancer cell lines was determined according to previously described procedures [67–68].
